# Predominance of gram-negative multidrug-resistant pathogens causing lower respiratory tract infections among gene X-pert negative presumptive tuberculosis patients in Dar Es Salaam, Tanzania

**DOI:** 10.1080/07853890.2025.2550577

**Published:** 2025-08-23

**Authors:** Irene Rabiel, Mtebe Majigo, Loveness Urio, Lilian Nkinda, Peter Richard Torokaa, Elieshiupendo M. Niccodem, Mabula Kasubi, Doreen Kamori, Joel Manyahi, Agricola Joachim

**Affiliations:** a Department of Epidemiology and Biostatistics, Muhimbili University of Health and Allied Sciences, Dar es Salaam, Tanzania; b Tanzania Field Epidemiology and Laboratory Training Program, Dar es Salaam, Tanzania; c Department of Microbiology and Immunology, Muhimbili University of Health and Allied Sciences, Dar es Salaam, Tanzania; d President’s Office Regional Administration and Local Government, Dodoma, Tanzania; e Department of Microbiology and Immunology Kilimanjaro Christian Medical Centre, Kilimanjaro, Tanzania; f Department of Diagnostic and Laboratory Services, Central Laboratory Pathology, Muhimbili National Hospital, Dar es Salaam, Tanzania

**Keywords:** Antibiotic susceptibility testing, GeneXpert, lower respiratory tract infection, presumptive tuberculosis, sputum quality

## Abstract

**Background:**

Lower respiratory tract infections (LTRIs) represent a significant global health burden. The clinical presentation of pulmonary tuberculosis (PTB) and other LRTIs often overlap, making it difficult to differentiate based on clinical features only. This study aims to investigate the role of other bacteria pathogens in LRTIs among presumptive TB patients and antibiotic susceptibility patterns for appropriate patient management.

**Materials and methods:**

We conducted a cross-sectional study among patients with symptoms and signs suggestive of PTB at Muhimbili National Hospital and Infectious Diseases Centre in Dar es Salaam, Tanzania. Sputum samples collected for TB diagnosis using the original GeneXpert system were investigated for other causes of LRTIs. The sputum samples were assessed for quality based on the Bartlett criteria before culture. We performed descriptive statistics to summarize the data.

**Results:**

We assessed 470 sputum samples, of which 317(67.4%) were of good quality. Of 317 samples, 21(6.6%) were *Mycobacterium tuberculosis* (MTB) positive by GeneXpert, while 126(39.7%) had 138 significant bacterial isolates other than MTB. *Pseudomonas aeruginosa* 44/99(44.4%) was the prominent Gram-negative bacteria isolated, followed by *Klebsiella pneumoniae* 22/99(22.2%). High rates of resistance was detected towards ampicillin (98%), penicillin (92%), and amoxicillin-clavulanic acid (65%). A high proportion of isolates, 71/138(51.4%) were multidrug resistant (MDR).

**Conclusion:**

This study revealed a high prevalence of LRTIs caused by non-TB pathogens, particularly MDR strains in presumptive TB. MTB was detected only in high‑quality sputum samples. The high resistance rate to commonly prescribed antibiotics for LRTIs called for further large-scale studies to guide and/or refine treatment guidelines and optimize patient care.

## Introduction

Lower respiratory tract infections (LRTIs) are acute illnesses with cough as the main symptom and at least one other symptom like sputum production, dyspnoea, wheezing, or chest discomfort [1]. An estimated 489 million incident cases and 2.4 million deaths due to LRTIs have been reported during the last three decades [2]. Lower respiratory infection (LRTI) ranked fifth among the top ten causes of death globally and is the leading cause of death among communicable diseases [3]. In clinical practice, patients presenting with symptoms of LRTIs for more than two weeks and seeking help from a health facility are commonly considered presumptive tuberculosis (TB) patients. The high TB suspicion index is due to its public health importance and significant global health challenge that necessitates an early diagnosis and treatment. Approximately 10.6 million new TB cases and 1.3 million deaths are reported annually [3].

Apart from *Mycobacterium tuberculosis* (MTB), other pathogens like bacteria, viruses, and fungi contribute substantially to the global burden of LRTIs and complicate the diagnosis and treatment efforts [4]. However, bacteria are the most commonly reported, including*, Klebsiella pneumoniae, Streptococcus pneumoniae, Pseudomonas aeruginosa, Acinetobacter* spp*, Escherichia coli, and Staphylococcus aureus* [5–7]. Previous studies conducted in Tanzania reported bacterial pneumonia prevalence rates of 20.4% and 34.3% [8,9]. Gram-negative bacteria such as *Klebsiella pneumoniae* and *Pseudomonas aeruginosa* were the most commonly identified pathogens, while *Streptococcus pyogenes* was the predominant gram-positive organism. The similarity of pulmonary TB and other causes of LRTIs makes it difficult to differentiate based on clinical diagnosis. Laboratory investigation is paramount to confirm the diagnosis and provide appropriate management [10]. GeneXpert has significantly improved the efficiency of TB diagnosis and detection of rifampicin resistance, particularly in resource-limited settings, by providing timely results and enabling prompt initiation of treatment [11,12]. Culture-based techniques remain the gold standard for identifying bacterial pathogens causing LRTIs. However, the yield of bacterial pathogens is also highly dependent on the quality of sputum samples, raising a need to evaluate the quality of sputum samples submitted for diagnosis of TB and LRTIs [10,11,13].

The emergence of antimicrobial resistance (AMR) presents a formidable challenge in treating bacterial LRTIs. The empirical use of antibiotics contributes to the emergence of AMR, particularly in low-income countries, where culture and antibiotic susceptibility testing are challenging in most health facilities [13,14]. Several studies have reported bacterial resistance to the commonly used antibiotics for treating LRTIs [5,14–16].

With a high TB burden, understanding the composition of pathogenic bacteria in sputum samples from TB-suspected patients is crucial for informing diagnostic and treatment protocols. This study aims to investigate the role of other bacteria pathogens in LRTIs among presumptive TB patients and antibiotic susceptibility patterns for appropriate patient management in our setting.

## Materials and methods

### Study design and setting

We conducted a cross-sectional study among patients with symptoms and signs suggestive of pulmonary TB. The study was conducted in Dar es Salaam, Tanzania, from March to June 2022 at Muhimbili National Hospital (MNH) and Infectious Diseases Centre (IDC). MNH is the largest tertiary hospital in Tanzania, attends a minimum of 1000 outpatients daily, and admits about 1000 to 1200 inpatients weekly. Approximately 40 patients with TB and 300 with presumptive TB are recorded monthly. IDC provides healthcare services for HIV, TB, and sexually transmitted infections. About 40 patients with TB attend the IDC TB clinic per month. The sputum samples collected at the two facilities were processed at the Central Pathology Laboratory (CPL) at MNH for TB diagnosis.

### Sample collection and transportation

Patients with signs and symptoms suggestive of pulmonary TB at outpatient clinics or admitted to the wards were provided with a sterile, wide-mouthed container and instructed by the attending clinician on collecting sputum. Within two hours of collection, sputum samples were immediately transported to CPL for processing.

### Sputum samples quality assessment

Sputum samples received at CPL were aliquoted into two portions: i) For MTB detection using the original GeneXpert test employing the older-generation of disposable cartridges (i.e. Xpert MTB/RIF) ii) For bacterial culture and susceptibility testing. Each sputum aliquot for culture was first assessed for quality based on the Bartlett criteria [17]. We assessed microscopically using a low power field, the number of neutrophils, the number of squamous epithelial cells and the presence of mucus strands. A total number of neutrophils per 10× field, less than 10 scored 0, 10–25 scored +1, and more than 25 scored +2. Squamous epithelial cells per 10× field less than 10 scored 0, 10–25 scored minus 1, and more than 25 scored minus 2. Sputum samples with mucus strands or mucoid, mucopurulent, purulent, or bloodstained scored +1. Sputum samples with a total score of +1 and above indicated that the sputum had good quality and met Bartlett’s inclusion criteria for sputum culture.

### Bacterial isolation and identification

Sputum samples were inoculated onto blood agar, chocolate agar, and MacConkey agar (MCA) plates (OXOID, UK). Blood agar and MCA plates were incubated in air, while chocolate agar plates were incubated under 5% CO_2._ All plates were incubated at 37 °C for 18–24 h. Bacterial pathogens were identified based on colonial morphological characteristics, Gram-staining reactions, and biochemical tests. Biochemical tests for Gram-positive cocci included catalase, coagulase, DNase, X and V disc, Optochin, and Bacitracin disc (Remel Europe Ltd, Dartford, UK). Gram-negative bacteria were identified using oxidase, citrate, urease, Kliger iron agar, sulphur indole, and motility (Oxoid Ltd, Hampshire, UK) [18]. Analytical Profile Index (API) 20E and API 20-NE (BioMérieux, France) tests were used to further identify Enterobacteriaceae and non-Enterobacteriaceae. Bacterial cultures were considered contaminated when the isolated organisms were part of the normal oral flora or consisted of environmental bacteria such as Bacillus species.

### Antimicrobial susceptibility testing

We used the Kirby Bauer disc diffusion method for antimicrobial susceptibility testing (AST) according to the Clinical and Laboratory Standard Institute (CLSI) guideline [19]. The following antibiotic discs were tested for gram-negative bacteria: ampicillin (10 µg), amoxicillin–clavulanate (20/10 µg), piperacillin–tazobactam (100/10 µg), cefepime (30 µg), ceftazidime (30 µg), ceftriaxone (30 µg), meropenem (10 µg), gentamycin (10 µg), amikacin (30 µg), and ciprofloxacin (5 µg). Aztreonam (30 µg) was used for *Pseudomonas aeruginosa* only. The antibiotic discs used for *Streptococci* species and, or *Staphylococcus* included penicillin (10 units), gentamycin (10 µg), ciprofloxacin (5 µg), erythromycin (15 µg), chloramphenicol (30 µg), trimethoprim–sulfamethoxazole (1.25/23.75 μg), clindamycin (2 µg), and doxycycline (30 µg). The isolates showing resistance to at least one antibiotic in three or more antimicrobial classes were defined as multidrug-resistant (MDR) [20].

We screened for methicillin resistance *Staphylococcus aureus* (MRSA) using a cefoxitin disc (30 μg) during AST. An inhibition zone of 21 mm for cefoxitin was considered MRSA. Clindamycin inducible resistance among *Staphylococcus aureus* isolates was detected using the D-test method according to CLSI guidelines [19]. Briefly, Erythromycin (15 µg) and clindamycin (2 μg) discs were placed 15–26 mm apart on the Muller Hinton Agar plate inoculated with *Staphylococcus aureus* and incubated aerobically for 16–18 h at 37 ^0^C. Flattening the zone of inhibition of clindamycin adjacent to the erythromycin disc (D-zone) was confirmed as inducible clindamycin resistance *Staphylococcus aureus*. The *Staphylococcus aureus* ATCC BAA-977 D-test positive and *Staphylococcus aureus* ATCC BAA-976 D-test negative were used as the controls.

Extended-spectrum-beta-lactamases (ESBL) screening was done using ceftazidime (30 μg) and ceftriaxone (30 µg), disc during AST, and interpreted according to the CLSI guideline [19]. Isolates with inhibition zones of ≤22 mm for ceftazidime and ≤25 mm for ceftriaxone were subjected to the disk combination method for phenotypic ESBL confirmation [19]. Isolates with an increased zone of inhibition of ≥5 mm between ceftazidime or cefotaxime (30 µg) disks with and without clavulanic acid were considered ESBL producers. A standard reference strain for positive ESBL-producing *K. pneumoniae* ATCC 700603 and negative ESBL-producing *E. coli* ATCC 25922 were used for quality control.

### Data analysis

Data analysis was performed using STATA version 15 Stata Corp. 2017. Descriptive statistics were summarized using frequency and proportions for categorical variables, whereas continuous variables were summarized with the measure of central tendency with respective measures of dispersion. A Pearson’s chi-square test was used to determine the differences between the two proportions. A *p* value <0.05 was used to declare statistical significance.

## Results

### Quality of sputum samples

**Figure 1.**
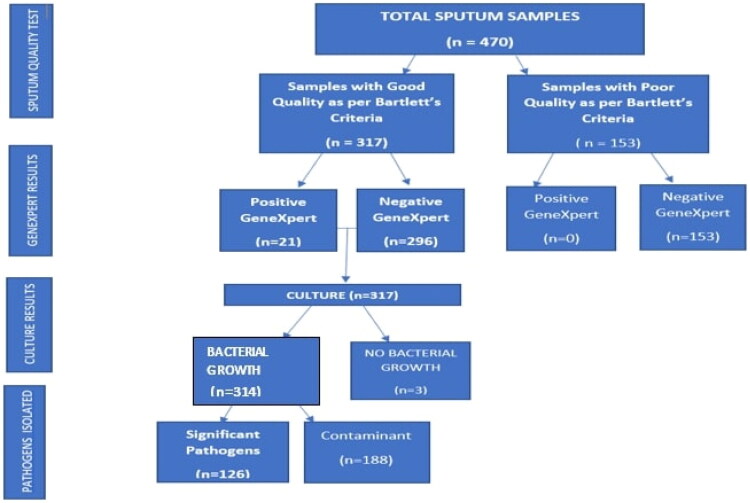
Flow chart showing the assessment of sputum samples for quality, the MTB GeneXpert test, bacterial culture, and pathogen isolation. MTB: *Mycobacterium tuberculosis.*

Using Bartlett’s criteria, we assessed the quality of 470 sputum samples submitted to CPL during the study period [17]. Two-thirds, 317(67.4%), were of good quality; 21/317 (6.6%) were MTB positive by GeneXpert. Samples that did not meet Bartlett’s criteria were 153; all were MTB negative by GeneXpert. MTB positive was 21/470 (4.5%) (Figure 1).

### Socio-demographic characteristics of the study participants

A total of 317 sputum samples were enrolled in this study to detect bacterial pathogens causing LRTI. The mean age was 37.6 (±15.5); nearly half, 154/317 (48.6%) were aged between 18 and 40. Most participants, 232/317 (73.2%), were males, and one-third, 106/317 (33.4%), were from Ilala municipality. Most samples, 285/317 (89.9%) were from MNH.

### Spectrum of causative agents of LRTI

**Figure 2.**
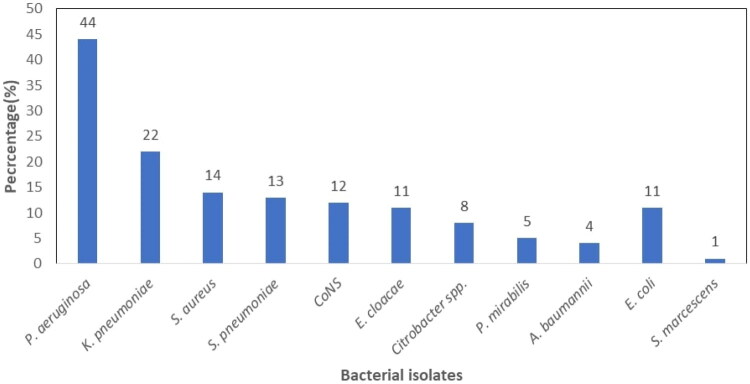
Bacteria isolated from cultures of 317 presumptive TB patients who submitted a good quality sputum specimen. TB: tuberculosis.

Of the 317 sputum samples cultured, 126 (39.7%) had significant bacterial growth other than MTB. Of these, 114/126 (90.5%) showed mono-bacterial pathogens, while 12/126 (9.5%) showed multi-bacterial pathogens, totalling 138 isolated bacterial pathogens. A small proportion of 5 (3.9%) had both MTB detected and other bacterial pathogens for LRTIs. Gram-negative bacteria were the predominant isolates 99/138 (71.7%) compared to Gram-positive cocci 39/138 (28.3%). Of the Gram-negative bacteria, *Pseudomonas aeruginosa* 44/99 (44.4%) was the most isolated bacteria, followed by *Klebsiella pneumoniae* 22/99 (22.2%), while *Staphylococcus aureus* 14/39 (35.9%) and *Streptococcus pneumoniae* 13/39 (33.3%) were the frequently isolated Gram-positive bacteria (Figure 2).

### Proportion of bacterial isolates among study participants

The proportion of significant pathogens isolated was 138/317 (43.5%). Patients aged >60 years had a higher proportion of pathogenic bacteria 8/17 (47.1%) than other age groups. Regarding gender, females had a high proportion of bacterial pathogens isolated 41/85 (48.2%) compared to males 85 (36.6%). A high proportion of bacterial pathogens, 21/46 (45.7%) isolated, came from Temeke compared to another district, though the difference was not statistically significant (*p* = 0.65). High frequency of bacterial pathogen causing LRTIs 118/285 (41.4%) were isolated from patients attending MNH (Table 1).

**Table 1.** Proportion of LRTI among 317 presumptive TB patients who submitted a good quality sputum specimen.

		Culture results		
Variable	Total, *N*	Positive, *N* (%)	Negative, *N* (%)	*P* value
Overall	317	126 (39.8)	191 (60.2)	
Age				
<17	17	6 (35.3)	11 (64.7)	0.792
18–40	154	58 (37.6)	96 (62.3)	
41–60	129	54 (41.9)	75 (58.1)	
>60	17	8 (47.1)	9 (52.9)	
Sex				
Female	85	41 (48.2)	44 (51.8)	0.062
Male	232	85 (36.6)	147 (63.4)	
Residence				
Ilala	106	40 (37.7)	66 (62.3)	0.656
Temeke	46	21 (45.7)	25 (54.4)	
Kinondoni	44	15 (34.1)	29 (65.9)	
Ubungo	32	11 (34.4)	21 (65.6)	
Others^a^	89	39 (43.8)	50 (56.2)	
Health facility				
MNH	285	118 (41.4)	167 (58.6)	0.072
IDC	32	8 (25.0)	24 (75.0)	

^a^Participants from outside Dar es Salaam, MNH Muhimbili National Hospital; IDC Infectious Disease Clinic.

### Antimicrobial resistance pattern

Overall, isolates showed the highest rate of resistance to ampicillin (98%), followed by amoxicillin–clavulanic acid (65%). For Gram-positive, high resistance was detected towards Penicillin (92%), while 58% were methicillin-resistant *Staphylococci* spp. A low rate of resistance was demonstrated against Amikacin (6%), Piperacillin/Tazobactam (13%), and Cefepime (14%) for gram-negative bacteria (Table 2).

**Table 2.** Antimicrobial resistance pattern of bacterial pathogen causing LRTI.

Isolates	*N*	% Antimicrobial resistance																		
		AMP	CN	CIP	CAZ	CTX	FEP	AMC	MEM	SXT	AK	TPZ	ATM	P	E	DA	DO	C	FOX	AZM
*P. aeruginosa*	44	ND	16	20	25	ND	5	ND	25	ND	ND	9	25	ND	ND	ND	ND	ND	ND	ND
*K. pneumoniae*	22	100	14	36	68	73	32	50	23	82	9	14	ND	ND	ND	ND	ND	ND	ND	ND
*E. cloacae*	11	91	27	36	73	91	27	91	73	9	0	18	ND	ND	ND	ND	ND	ND	ND	ND
*Citrobacter* spp	8	100	0	25	25	38	13	63	0	13	0	25	ND	ND	ND	ND	ND	ND	ND	ND
*P. mirabilis*	5	100	80	60	80	80	0	80	60	60	0	0	ND	ND	ND	ND	ND	ND	ND	ND
*A. baumannii*	4	ND	25	50	100	50	50	ND	0	50	0	0	ND	ND	ND	ND	ND	ND	ND	ND
Other GNB^a^	5	100	20	40	60	60	0	40	20	40	20	40	ND	ND	ND	ND	ND	ND	ND	ND
*S.pneumoniae*	13	ND	ND	ND	ND	ND	ND	ND	ND	100	ND	ND	ND	ND	38	15	31	0	ND	31
*S. aureus*	14	ND	36	36	ND	ND	ND	ND	ND	7	ND	ND	ND	93	29	7	21	100	29	ND
CoNS	12	ND	25	58	ND	ND	ND	ND	ND	25	ND	ND	ND	92	25	17	25	ND	92	ND
**Total**	**138**	**98**	**21**	**34**	**48**	**69**	**14**	**65**	**28**	**47**	**6**	**13**	**25**	**92**	**31**	**13**	**26**	**52**	**58**	**31**

^a^Other GNB: Gram negative bacteria include; *S. marcescens* and *E.coli*.

N: Number of isolates, ND: Not done, AMP: Ampicillin, CN: Gentamicin, CIP: Ciprofloxacin, CAZ: Ceftazidime, CTX: Cefotaxime, AMC: Amoxicillin/Clavulanic acid, MEM: Meropenem, AK: Amikacin, SXT: Trimethoprim/Sulfamethoxazole, FEP: Cefepime, TZP: Piperacillin/Tazobactam, ATM: Aztreonam, P: Penicillin-G, E: Erythromycin, DA: Clindamycin, DO: Doxycycline, C: Chloramphenicol, FOX: Cefoxitin, AZM: Azithromycin. CoNS: Coagulase negative staphylococci.

### Multidrug resistance bacterial pathogens causing LRTI

More than a quarter, 16/55 (29.1%) of the gram-negative pathogens were ESBL-producers, with *Klebsiella pneumoniae* having the highest proportion, 10/16 (62.5%). MRSA were detected in 4/14 (28.6%). More than half of the bacteria isolated from the presumptive tuberculosis patient were MDR, 71/138 (51.4%) (Table 3).

**Table 3.** Proportion of multidrug resistance bacterial pathogens isolated from cultures of 317 presumptive TB patients who submitted a good quality sputum specimen.

Bacterial isolates	*N*	Frequency	%
*P. aeruginosa*	44	8	18.2
*K. pneumoniae*	22	18	81.8
*Escherichia coli*	11	1	9.1
*Enterobacter cloacae*	11	9	81.8
*Acinetobacter. baumannii*	4	2	50.0
*Citrobacter freundii*	8	2	25.0
*Proteus mirabilis*	5	5	100.0
*S. mercescens*	1	1	100.0
*S. aureus*	14	11	78.6
*S. pneumoniae*	13	4	30.8
Coagulase negative staphylococci	12	10	83.3
**Total**	138	**71**	**51.4**

## Discussion

This study aimed to identify the aetiological agents and antimicrobial susceptibility pattern of LRTIs other than TB among suspected TB cases. A high proportion of the sputum samples (67.4%) submitted for diagnosis of MTB by Gene Xpert had good quality for aerobic bacterial culture. However, only 6.6% of these samples tested MTB positive, while a high proportion (93.4%) detected MTB negative results. Notably, none of the samples deemed poor quality (32.6%) tested positive for MTB. The study revealed a high prevalence of LRTIs (39.8%) among individuals suspected of having TB, primarily caused by pathogenic bacteria other than MTB. Alarmingly, a substantial proportion of these bacterial isolates were multidrug-resistant. This highlights a significant public health concern as it limits treatment options and increases the risk of treatment failure, prolonged illness, and higher mortality. MDR infections often require expensive and potentially more toxic therapies. The high MDR rate underscores the need for stronger antibiotic stewardship and surveillance.

The prevalence of positive MTB cases in this study (6.6% by GeneXpert) was comparable to findings from a study conducted two years ago in Mwanza, Tanzania (7.2%) [5]. However, our finding on the prevalence of MTB was lower than that of an earlier study from Mwanza, Tanzania, in adults (25.0%) and children (15%) [16]. Another study done in Cambodia reported a prevalence of 19% [21]. The low TB prevalence observed in the current study may be attributed to several factors, including differences in study populations and diagnostic methodologies.

The quality of sputum samples for diagnosing pulmonary TB or bacterial pneumonia plays a vital role by increasing the MTB detection rate using GeneXpert and the bacterial culture yielding rate. In the current study, the quality of the sputum samples submitted to the laboratory was also evaluated using Bartlett’s criteria, with (67.4%) having good quality. MTB (6.6%) was detected in sputum samples from TB suspected cases with good quality, while none was detected in sputum samples with poor quality. This indicates the importance of adequately instructing the patients on collecting quality samples to avoid misdiagnosis or delayed diagnosis, leading to poor health outcomes due to inappropriate treatment. Establishing standardized criteria for assessing sample quality before processing is essential to improve diagnostic accuracy. Notably, sputum samples with >10 neutrophils/LPF was good indicators for diagnosing pulmonary TB [13].

A noteworthy observation in this study was the high rate of negative MTB detection by GeneXpert despite clinical presentations suggesting pulmonary TB, indicating potential infections by other pathogenic bacteria. The prevalence of non-TB pathogenic bacteria in TB-suspected patients in this study was 39.7%, which is higher than the prevalences of 16.3% and 34.3% reported in previous studies in Mwanza, Tanzania [5,8]. The high prevalence in our study could be attributed to the fact that only sputum samples with good quality, as per Bartlett’s criteria, were subjected to culture. On the other hand, our findings are slightly lower than those of the study done in Cambodia, which reported a prevalence of 44% [21], and Nepal (47.0%) [14]. The difference could be that our study included only presumptive TB cases, while other studies enrolled both TB and other bacterial cases. Prior antibiotic use might have also reduced the bacterial growth rate and the difference in the population studied.

Multi-microbial infections were detected in a small proportion (3.2%) of sputum samples, underscoring the complexity of LRTIs and the need for comprehensive diagnostic approaches. A similar finding has been observed in the study of TB-suspected cases [22]. *Pseudomonas aeruginosa* emerged as the predominant pathogen, followed by *Klebsiella pneumoniae,* which is consistent with previous studies in Mwanza, Tanzania, and Nigeria [5,23]*. Staphylococcus aureus* and *Streptococcus pneumoniae* were the frequently encountered gram-positive pathogenic bacteria. A similar pattern has been reported in other studies conducted in Tanzania [5].

In the current study, we observed a high resistance to ampicillin (98%), third-generation cephalosporin (48–69%), and amoxicillin–clavulanic acid (65%). Moderate resistance was observed to trimethoprim/sulfamethoxazole (47%), ciprofloxacin (34%), and azithromycin (31%) (*Streptococcus pneumoniae)*. These are among the antibiotics recommended for treating LRTI by the standard treatment guidelines and national essential medicines list for Tanzania Mainland [24]. This raises concern about the effectiveness of the antibiotic’s clinicians prescribe in treating LRTI in our setting. Thus, samples with GeneXpert negative results should be subjected to culture to isolate other pathogenic bacteria apart from MTB and antimicrobial susceptibility testing for appropriate antibiotic treatment.

This study observed ESBL in 29% of the Gram-negative bacteria, slightly higher than what was reported in Nepal (25.6%) [25]. Furthermore, this study revealed nearly similar rates of MDR (51.4%) compared to previous studies (55.6%) [25,26]. The observed difference could be attributed to the empirical treatment given to patients, especially patients with GeneXpert negative results. The emergence of ESBL producers and MDR strains highlights the urgent need for culture and antimicrobial susceptibility testing to guide appropriate antibiotic treatment. Large-scale multi-site studies are needed to gather enough data to guide patient management. Such data will also guide policymakers in revising the national standard treatment guidelines for LRTIs. On the other hand, amikacin, cefepime, and piperacillin/tazobactam demonstrated low rates of AMR, suggesting that these antibiotics can be used to treat LRTIs. Whenever possible, screening for other causes of LRTI, like viruses and difficult-to-culture pathogenic bacteria, should be overemphasized, especially in culture-negative among TB presumptive cases.

The study had limitations, including incomplete data on antibiotic use history, which might have affected the yielding rate. Furthermore, the lack of HIV/AIDS status warrants consideration in interpreting the findings.

## Conclusion

This study found a high prevalence of LRTIs caused by non-TB pathogens, particularly MDR strains, among individuals suspected of having TB. MTB was detected only in high‑quality sputum samples using the GeneXpert. The alarmingly high resistance rate to commonly prescribed antibiotics for LRTIs called for further large-scale studies to guide and/or refine treatment guidelines and optimize patient care. Screening for alternative causes of LRTIs should be prioritized, especially in culture-negative TB presumptive cases.

## Data Availability

The information and data analysed in this research are available from the corresponding author and can be shared upon rational request.
